# Effects of Lactic Acid Bacteria Inoculants on Fermentation Quality, Bacteria Communities and Antibiotic Resistance Genes in Whole-Crop Corn Silage

**DOI:** 10.3390/microorganisms13091977

**Published:** 2025-08-25

**Authors:** Dandan Chen, Dan Yang, Tianxin Guo, Qing Zhang

**Affiliations:** 1 Shandong Key Laboratory for Germplasm Innovation of Saline-Alkaline Tolerant Grasses and Trees, College of Grassland Science, Qingdao Agricultural University, Qingdao 266109, China; cdd1744301540@163.com; 2 College of Forestry and Landscape Architecture, South China Agricultural University, Guangzhou 510642, China; ydycbwywtb@163.com (D.Y.); g18028703192@163.com (T.G.)

**Keywords:** antibiotic resistance genes, whole-crop corn silage, lactic acid bacteria, mobile genetic elements, bacterial communities

## Abstract

Feed is an important source of antibiotic resistance genes (ARGs) in animals and products, posing significant potential risks to human health and the environment. Ensiling may present a feasible method for reducing ARGs in animal feed. This study involved the addition of four types of lactic acid bacteria (LAB) inoculants, *Lactiplantibacillus plantarum* (LP), *Pediococcus acidilactici* (P), *Enterococcus faecium* (E), and *Ligilactobacillus salivarius* (LS), to whole-crop corn silage to investigate changes in ARGs, mobile genetic elements (MGEs), and their transmission risks during ensiling. The results indicated that the addition of LAB significantly reduced the ammonia nitrogen content and pH value of whole-crop corn silage, inhibited the growth of harmful microorganisms, and increased the lactic acid content (*p* < 0.05). The improvement effect was particularly pronounced in the P treatment group. Natural fermentation plays a significant role in reducing ARG abundance, and the addition of different types of lactic acid bacteria helps reduce the abundance of both ARGs and MGEs. Specifically, the LS treatment group exhibited a significant decrease in MGE abundance, potentially reducing the horizontal transmission risk of ARGs. Furthermore, variations in ARG abundance within different LAB strains were detected, showing a consistent trend with that in silage. ARGs and MGEs were correlated with the fermentation parameters and microbial communities (*p* < 0.05). This suggests that adding LAB with low levels of ARGs to silage can effectively reduce ARG contamination. Bacterial community structure, MGEs, and fermentation quality may act as driving forces for the transfer and dissemination of ARGs in the silage ecosystem.

## 1. Introduction

The application of antibiotics plays a crucial role in safeguarding human health and enhancing livestock productivity [1]. However, due to the lack of relevant regulatory systems and people’s indispensable reliance on antibiotics, the usage of antibiotics has increased sharply. After humans or animals use antibiotics, only a small portion of cfu/g is absorbed and utilized, with the majority being excreted into the environment, ultimately leading to ecological pollution [2]. While the residual antibiotics in the environment do not directly harm humans, an increasing number of microbes are developing antibiotic resistance and carrying antibiotic resistance genes (ARGs). These genes may have more severe effects on the environment than the antibiotics themselves [3]. Currently, numerous studies indicate that the interactions between humans, animals, food, and the environment are the main pathways through which ARGs and their potential host bacteria spread to agricultural ecosystems. ARGs can move into plants via soil through microbial transfer, and bacteria carrying ARGs can colonize plants in various ways, eventually transmitting through the food chain to humans [4,5].

Silage is a method of anaerobic microbial fermentation used to preserve fresh forage. For a long time, silage has been valued for its aromatic, succulent, palatable, and nutritionally rich characteristics, enhancing animals’ appetite and digestive function. It effectively addresses the issue of balanced feed supply in spring and winter, becoming a primary source of roughage for ruminants [6,7]. However, studies suggest that ARGs may transfer to plants from contaminated soil, wastewater, and feces via rhizosphere or phyllosphere microbiota, implying that silage could potentially serve as a source of ARGs [4,8]. Although the presence of ARG in corn silage has been confirmed, the specific transmission mechanism and abundance changes of ARG in the entire corn silage plant are still unclear.

Lactic acid bacteria (LAB) refers to a group of Gram-positive cocci or rods capable of fermenting carbohydrates to produce abundant lactic acid [9]. LAB, well-known as probiotics, are commonly used as commercial starter cultures and play a vital role in silage fermentation. Some studies have found that lactic acid bacteria harbor ARGs, and strains with antibiotic resistance may potentially transmit to other bacteria in the human gastrointestinal tract, posing a threat to human health [10,11]. Intriguingly, it was discovered through metagenomics that inoculating alfalfa silage with *Lactobacillus plantarum* MTD-1 or *Lactobacillus buchneri* 40788 could reduce the absolute abundance of ARGs by decreasing the levels of host bacteria and plasmids [12]. Xu et al. (2021) indicated that after whole-crop corn silage fermentation, *Escherichia coli* and *Staphylococcus*, common pathogens, were significantly suppressed, possibly due to the dominance of lactic acid bacteria produced during the silage process, inhibiting pathogenic bacteria carrying ARGs and thereby reducing the relative abundance of ARGs [13]. In conclusion, the addition of lactic acid bacteria to silage feed plays a role in the abundance of ARGs, but the variations in ARG abundance when different types of lactic acid bacteria are added remain unexplored.

Therefore, this study investigates the influence of various types of lactic acid bacteria on ARG abundance in whole-crop corn silage using real-time PCR methods. The objectives of this study were (1) to investigate the abundance variation of ARGs and fermentation quality in whole-crop corn silage inoculated with different types of lactic acid bacteria; (2) to explore the factors influencing the abundance of ARGs in whole-crop corn silage; (3) to identify potential hosts of ARGs. It provides a feasible approach to control the ecological risks of ARGs in silage.

## 2. Materials and Methods

### 2.1. Silage Preparation

Whole-crop corn at the milk stage was collected from Zengcheng teaching and research bases of South China Agricultural University (N 23°14′, E 113°38′, Guangzhou, China). The whole-crop corn material was chopped to 2 cm in the laboratory. The chopped whole-crop corn was mixed well and then inoculated with distilled water (control, CK), *Lactiplantibacillus plantarum* (LP, 1 × 10^6^ cfu/g fresh matter), *Pediococcus acidilactici* (P, 1 × 10^6^ fresh matter), *Enterococcus faecium* (E, 1 × 10^6^ cfu/g fresh matter), and *Ligilactobacillus salivarius* (LS, 1 × 10^6^ cfu/g fresh matter) separately. After thorough mixing, it was packed into polyethylene plastic bags (20 cm × 30 cm, Dongguan Bojia Packaging, Dongguan, China) (approximately 150 g of each bag, 6 bags for each treatment), and vacuumed, sealed by a vacuum-sealing machine. Subsequently, all silage samples were stored at room temperature (27 °C to 32 °C) and opened after 30 days of fermentation.

### 2.2. Fermentation Characteristics and Chemical Compositions Analyses

Silage fermentation parameters were determined according to our previous study [14]. First of all, 20 g of fresh samples were collected and added to 180 mL of pure water in a refrigerator at 4 °C for full immersion, filtered after 24 h, and the pH values were determined using a Raymag PHS-3C pH meter (Shanghai Precision Scientific Instrument Co., Ltd., Shanghai, China), after which organic acids were measured using High Performance Liquid Chromatography system (column: Shodex Ionpak KC-811, Showa Denko K.K., Kawasaki, Akashi, Japan; detector: DAD, 210 nm, SPD-20A, Shimadzu Co., Ltd., Kyoto, Japan; eluent: 0.1% HP_3_O_4_/H_2_O, 1.0 mL/min; temperature: 40 °C) and ammonia nitrogen was analyzed using the phenol-hypochlorite colorimetric method. After that, another 20 g of silage and 180 mL of sterilized saline were homogenized and serially diluted. Lactic acid bacteria, coliforms, and yeasts were cultured using MRS agar plates, violet red bile agar (VRBA) plates, and Rose Bengal agar plates, respectively (Guangzhou Huankai Microbial Technology Co., Ltd, Guangzhou, China). Eventually, the remaining samples were dried in a constant temperature oven at 65 °C for 48 h to measure the dry matter content. The true and crude protein content was analyzed using a fully automated Kjeldahl nitrogen analyzer (Kjeltec 8400, FOSS, Denver, CO, USA) according to Wang et al. (2019), where non-protein nitrogen was calculated by the discrepancy between them [15].

### 2.3. DNA Extraction

DNA extraction was performed from whole-crop corn silage samples (10 g) using the HiPure Bacterial DNA Kit (Magen, Guangzhou, China). A total of 36 DNA samples were collected, and the purity and concentration of extracted DNA were determined by agarose gel electrophoresis and a NanoDrop ND-2000 spectrophotometer (Thermo Fisher Scientific, Wilmington, DE, USA), respectively. The DNA results were detected on 1% agarose gel, and the successfully extracted DNA was immediately placed in a refrigerator at −20 °C to facilitate the subsequent detection of ARGs, MGEs, and microbial community structure.

### 2.4. Quantification of ARGs and MGEs

A total of 8 species of ARGs and 3 species of MGEs were detected, including sulfonamide resistance genes (*sul1* and *sul2*), macrolide resistance genes (*ermB*), methotrexate resistance genes (*dfrK*), quinolone resistance genes (*oqxB*), multiple drug resistance regulatory genes in *Escherichia coli* (*acrR*), multidrug transporter resistance genes (*mexA*), fluoroquinolone resistance genes (*aac(6′)-ib-cr*), and MGEs (*Intl1*, *ISCR1*, and *Tn916/1545*). The experimental procedure refers to the study of Sanz et al. (2022) [16]. Briefly, the plasmid was amplified by ordinary PCR and gel recovery, then constructed, and finally detected by qPCR. Specific primer sequences and annealing temperatures are shown in Table 1. The qPCR mixture consisted of 10 μL of 2 × SYBR Green qPCR Premix, 0.4 μL of pre- and post-primers, and 1 μL of DNA template, with 8.2 μL of nuclease-free water to make the final volume of 20 μL. The qPCR procedure consisted of initial deformation at 95 °C for 30 s, followed by 40 cycles of denaturation at 95 °C for 10 s, and annealing and extension at 60 °C for 30 s. The specific primer sequences and annealing temperatures are provided in the Table 1.

### 2.5. Microbial Diversity Analysis

The extracted DNA was used to amplify the V3-V4 region of 16s rRNA with primers 341F (CCTACGGGGNGGGCWGCAG) and 806R (GGACTACHVGGGGTATCTAAT). The purified polymerase chain reaction (PCR) products were sequenced using an Illumina HiSeq 2500 system (Illumina, San Diego, CA, USA). Finally, microbial communities were analyzed for α-diversity, β-diversity, and relative abundance via a free online platform (http://www.omicshare.com/tools).

### 2.6. Date and Statistical Analysis

Data were statistically analyzed using one-way analysis of variance (ANOVA) in SPSS 24.0 to assess the effect of LAB on fermentation characteristics. Duncan’s method of extreme variance was used for multiple comparisons of differences between the means of individual samples, and a significant effect was inferred if the *p*-value was less than 0.05, indicated by a different indicated by a different alphabet. Box line plots of the abundance of ARGs and MGEs were were performed using GraphPad Prism 8 and Adobe Illustrator CC2018. Microbial community correlation maps were obtained using the (https://www.omicsmart.com/#/project, accessed on 2 December 2023). Network diagram analysis was performed using Gephi software 64.

## 3. Results

### 3.1. Fermentation Characteristics and Chemical Compositions of Whole-Crop Corn Silage

Fermentation characteristics and chemical compositions of whole-crop corn silage with or without LAB are listed in Table 2. During fermentation, the pH of all samples was below 4, with a significant decrease (*p* < 0.05) observed in the LP-treated group. The addition of LAB had a significant impact on the content of lactic acid, acetic acid, and propionic acid. Compared to the control group, the P-treated group showed a significant increase (*p* < 0.05) in the content of lactic acid, acetic acid, and propionic acid, while the LP and LS-treated groups exhibited a significant decrease (*p* < 0.05) in the content of lactic acid and acetic acid. No butyric acid was detected in this experiment. At the same time, inoculation with these four strains of lactic acid bacteria increased (*p* > 0.05) the CP content of the whole-crop corn silage and significantly reduced (*p* < 0.05) the content of NH_3_-N. Among them, the P-treated group performed best in improving the fermentation quality of the whole-crop corn silage.

### 3.2. Abundance Changes in ARGs and MGEs During Silage

**Figure 1:**
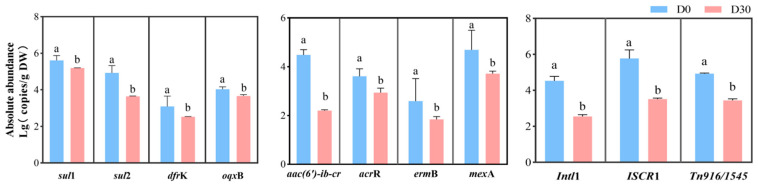
Abundances of ARGs and MGEs in whole-crop corn before and after ensiling. DW, dry weight; D0, fresh whole-plant corn; D30, whole-plant corn after 30 days of silage. Values with dissimilar superscript letters between groups represent a significant difference in the 5% Tukey test.

Overall, the absolute abundance of different types of ARGs and MGEs was lower (*p* < 0.05) in whole-crop corn after 30 days of ensiling than before ensiling (Figure 1). Specifically, prior to ensiling, the highest absolute abundance was observed for *ISCR1* at 5.77 logs, while the lowest was for ermB at only 2.59 logs. Among these ARGs and MGEs, the removal rate of *aac(6′)-ib-cr* was as high as 50.89%, followed by intl1, *ISCR1*, *Tn916/1545*, and *erm*B, with removal rates of 43.71%, 39.17%, 30.22%, and 28.96%, respectively. In contrast, *sul1*, *oqxB*, and *acrR* had lower removal rates of 7.49%, 9.41%, and 18.33%, respectively.

### 3.3. Effect of LAB on the Removal of ARGs and MGEs in Silage

**Figure 2:**
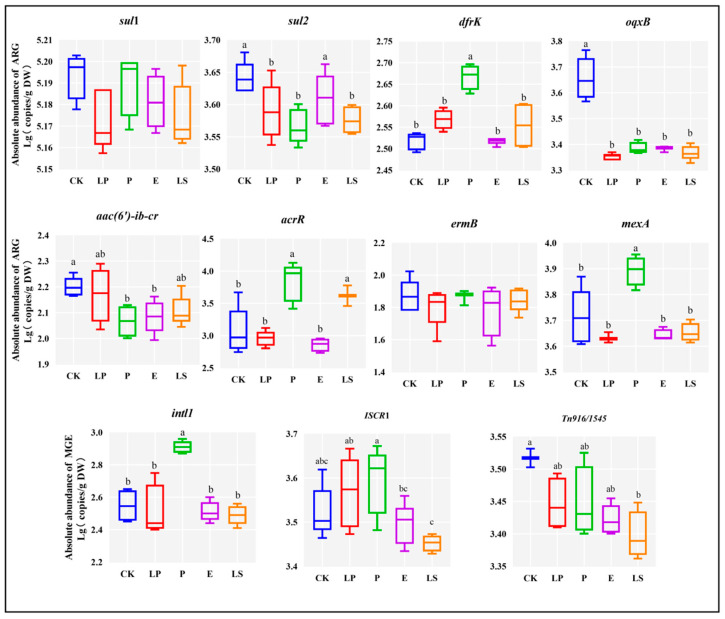
Changes in ARGs and MGEs in whole-plant corn silage after 30 days of fermentation. DW, drained weight; CK, the control; LP, *Lactiplantibacillus plantarum*; P, *Pediococcus acidilactici*; E, *Enterococcus faecium*; LS, *Ligilactobacillus salivarius*. Values with dissimilar superscript letters between groups represent a significant difference in the 5% Tukey test.

As shown in Figure 2, lactic acid bacteria strains showed influence on the abundance of ARGs and MGEs. LP reduced (*p* < 0.05) the abundance of *sul2* and *oqxB*. Inoculation with P significantly reduced (*p* < 0.05) the abundance of *sul2* and *aac(6′)-ib-cr*, with removal rates of 27.52% and 53.88%, respectively. Similarly, inoculation with LS markedly decreased the abundance of *ISCR*1 and *Tn916/1545*, with removal rates of 40.17% and 31.39%, respectively. Compared to the CK, *acrR*, *intl1*, *dfrK*, and *mexA* significantly increased in the P treatment group, while ISCR1 significantly increased under LP and P treatment (*p* < 0.05). MGEs can facilitate the transfer of ARGs, and the abundance of MGEs was also influenced by lactic acid bacteria strains. Specifically, intl1 increased after inoculation with P, and LS reduced the abundance of *Tn916/1545*.

### 3.4. ARGs and MGEs in Lactic Acid Bacteria Strains

**Figure 3:**
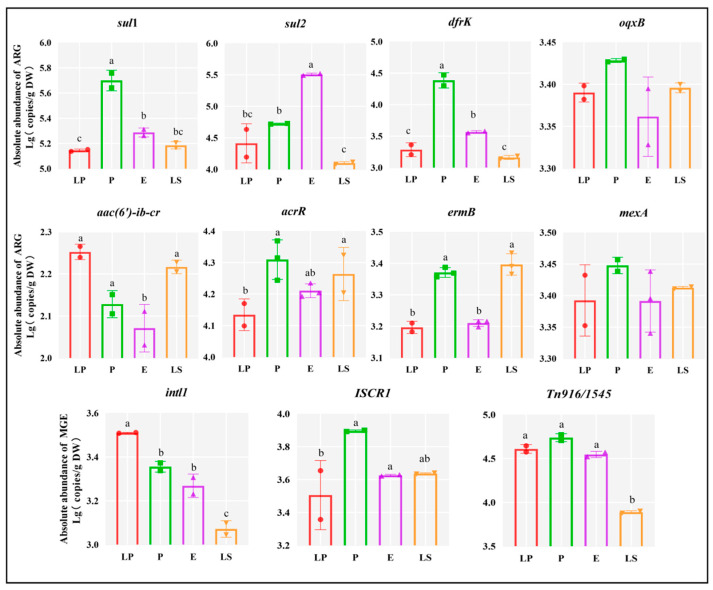
Abundance of ARGs and MGEs in lactic acid bacteria strains. DW, dry weight; LP, *Lactiplantibacillus plantarum*; P, *Pediococcus acidilactici*; E, *Enterococcus faecium*; LS, *Ligilactobacillus salivarius*. Values with dissimilar superscript letters between groups represent a significant difference in the 5% Tukey test.

Overall, this experiment conducted quantitative analysis of lactic acid bacteria inoculants and found significant differences in the absolute abundance of various types of ARGs and MGEs, except for *oqxB* and *mexA* (*p* < 0.05) (Figure 3). With the exception of *intl1*, the trends in the abundance of resistance genes in the lactic acid bacteria were generally consistent with the changes in ARGs and MGEs in silages after inoculation with corresponding lactic acid bacteria strains. LP and LS showed the lowest (*p* < 0.05) abundance of *sul1*, *sul2*, and *dfrK*. P and LS exhibited the highest (*p* < 0.05) abundance of *acrR* and *ermB*. LS showed lower (*p* < 0.05) intl1 and *Tn916/1545* than other strains.

### 3.5. Changes in the Bacterial Community Structure of Silage After LAB Treatment

#### 3.5.1. Bacterial Alpha Diversity

Microbial alpha diversity across treatments was evaluated using OTUs (Sobs), richness (Chao and Ace indices), and diversity (Simpson index) (Table 3). After 30 days of fermentation, Sobs, Chao, and Ace values decreased while the Simpson index increased in whole-crop corn silage, suggesting a decrease in microbial community richness but an increase in diversity. After the addition of LAB, the Chao and Ace values, as well as the Simpson index of whole-crop corn silage in the LP treatment group, were significantly higher compared to the CK group. This indicates that the addition of LP can enhance alpha diversity in silage.

#### 3.5.2. Microbial Community Structure

**Figure 4:**
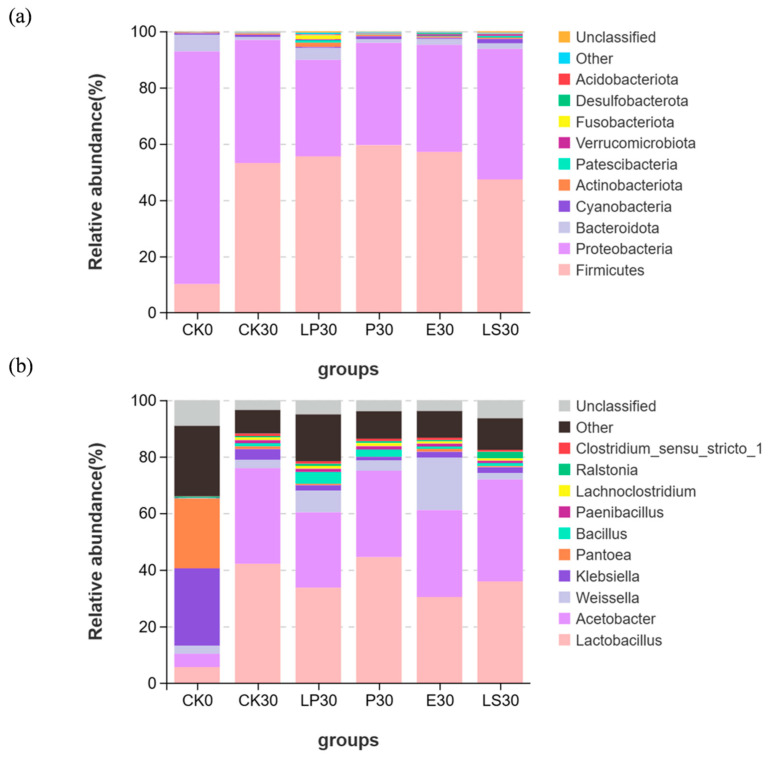
Bacterial communities in whole-plant corn silage with or without LAB strains. (**a**) Phylum level, (**b**) genus level. CK0, whole-crop corn before ensiling; CK30, silage without LAB strains after 30 days of fermentation; LP30, silage treated with *Lactiplantibacillus plantarum* after 30 days of fermentation; P30, silage treated with *Pediococcus acidilactici* after 30 days of fermentation; E30, silage treated with *Enterococcus faecium* after 30 days of fermentation; LS30, silage treated with *Ligilactobacillus salivarius* after 30 days of fermentation.

The microbial community structures at the phylum level for each group are depicted in Figure 4 based on the species annotation results of the 16S rRNA. Throughout the entire experiment, the dominant phyla across groups were Firmicutes, Proteobacteria, and Bacteroidota. At day 0 of ensiling, Proteobacteria dominated in whole-crop corn with a relative abundance of 82.69%, followed by Firmicutes and Bacteroidota at 10.15% and 5.93%, respectively. After 30 days of ensiling, the dominant phyla in the CK group shifted to Firmicutes and Proteobacteria, accounting for 46.59–57.72% and 37.62–51.19% in relative abundance, respectively. In different lactic acid bacteria strain treatments, Firmicutes and Proteobacteria maintained dominance. Relative abundances of Firmicutes significantly increased by 2.36%, 4.20%, and 6.34% in the LP, E, and P treatment groups, respectively, while decreasing by 5.84% in the LS group compared to CK. Proteobacteria relative abundances significantly increased by 2.69% in the LS treatment group but decreased by 5.61%, 7.35%, and 9.40% in the E, P, and LP treatment groups, respectively. Prior to ensiling, the dominant genera in the CK group were *Klebsiella* (27.39%), *Pantoea* (24.62%), and *Lactobacillus* (5.63%). After 30 days, the predominant genera were *Lactobacillus* (42.09%), *Acetobacter* (33.83%), and *Weissella* (2.92%). With the addition of LAB, compared to the CK group, the relative abundance of *Lactobacillus* decreased in the E group, but *Weissella*’s relative abundance increased significantly by sixfold. In the P group, the relative abundance of *Lactobacillus* increased significantly by 2.35%, while *Bacillus* increased by 2.43%. In the LP group, the relative abundances of *Bacillus* and *Weissella* increased by 3.03% and 4.71%, respectively.

### 3.6. Relationships Between Silage Quality, ARGs, and the Bacterial Community Structure

**Figure 5:**
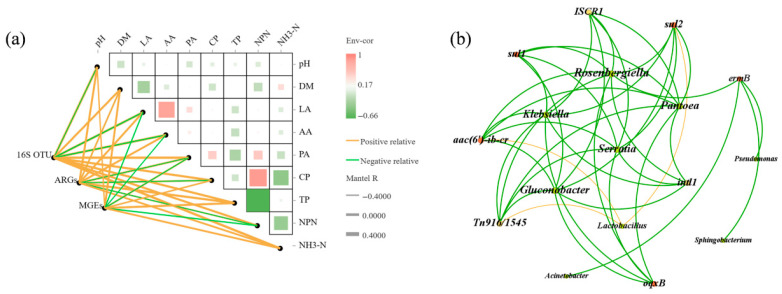
Mantel test of 16s OTUs, ARGs, MGEs, and fermentation characteristics (**a**). The size of the square area represents the magnitude of the correlation and the different colors of the squares represent positive or negative correlation. The color and width of the edges indicate statistical significance and Mantel’s r statistic (**b**). Nodes represent ARGs, MGEs, and bacteria at the genus level, and connections represent significant correlations between them (*p* < 0.05). The bigger nodes indicate higher correlation.

The Mantel test examined the relationships between microbial communities, ARGs, MGEs, and fermentation quality indicators to investigate the mechanism by which lactic acid bacteria reduce ARGs (Figure 5a). From Figure 5, it is evident that fermentation quality has a certain impact on ARGs and MGEs. Among these correlated fermentation qualities, ARGs exhibited a positive correlation with pH, DM, LA, AA, TP, and NH3-N content, and a negative correlation with PA and CP content. MGEs showed a positive correlation with pH, DM, CP, TP, and NH_3_-N content, and a negative correlation with LA and AA content. Based on Pearson correlation coefficients, network analysis was employed to examine the co-occurrence of ARGs and their potential host bacteria (Figure 5b). The network analysis revealed 17 nodes and 42 edges, including 5 ARGs, 3 MGEs, and 9 bacterial genera (R > 0.8, *p* < 0.05). These nine bacterial genera represent potential hosts for the ARGs, with most belonging to Proteobacteria. Pantoea showed a positive correlation with *sul1*, *sul2*, *oqxB*, *ISCR1*, *intl1*, *aac(6′)-ib-cr*, and *Tn916/1545*, suggesting that Pantoea may be the potential host for these genes. In contrast, *Lactobacillus* displayed a negative correlation with *sul2*, *Tn916/1545*, and *aac(6′)-ib-cr*, indicating that *Lactobacillus* is not their potential host. Notably, all MGEs were closely associated with their potential host bacteria.

## 4. Discussion

Ensiling is a microbiological anaerobic fermentation process to preserve fresh pasture [17]. It mainly uses lactic acid bacteria naturally attached to the surface of the plant to break down the soluble carbohydrates in the plant and produce organic acids that cause a rapid drop in pH and inhibit the growth and reproduction of harmful microorganisms [18]. Whole-crop corn harbors abundant surface microbiota and contains relatively high levels of water-soluble carbohydrates [19]. It experiences minimal constraints during the fermentation process and exhibits high energy and favorable fermentation characteristics [20]. As a result, whole-crop corn silage serves as a primary roughage source in the rations of ruminant animals in many regions worldwide [21]. pH value serves as a crucial indicator reflecting the fermentation quality of silage. Studies have indicated that a pH value below 4.2 is the standard for high-quality forage [22]. Furthermore, lactic acid, a key metabolic product of lactic acid bacteria, is a major factor responsible for pH reduction. In this study, regardless of the addition or exclusion of lactic acid bacteria, the pH value was below 4.2. In comparison to the control group, the P treatment group exhibited a significant increase in lactic acid content, providing a favorable condition for silage fermentation. NH_3_-N content, known as an indicator of protein degradation in silage, significantly decreased after lactic acid bacteria inoculation. This might be because inoculants suppressed harmful microorganisms and reduced protein loss during the fermentation process [23]. The accelerated accumulation of lactic acid by the lactic acid bacteria likely contributed to a decrease in pH, thereby inhibiting the hydrolysis of proteins by detrimental microorganisms [24,25]. Consequently, the fermentation quality of corn silage was improved.

The quality and safety of whole-crop corn silage are closely related to the development of animal husbandry and human health [26]. Most of the ARGs detected in whole-crop corn silage belong to the fluoroquinolone, macrolide, and sulfonamide classes [27]. According to FDA classification, fluoroquinolones and macrolides are the most widely used drugs in global human medicine. Resistance genes of macrolides and fluoroquinolones are frequently detected in livestock feces [28,29]. For example, sulfonamides are commonly used in livestock breeding to prevent and treat infectious bacterial diseases like diarrhea [30]. Fluoroquinolones have a broad antibacterial spectrum and potent antibacterial effects, significantly enhancing bactericidal efficiency against intestinal bacteria in clinical use [31]. Macrolides and lincosamides are common medications in European cattle production for treating various infections [8]. This study found that these ARGs were all detected in whole-crop corn and reduced after 30 days of fermentation. It indicates that ensiling might be a feasible way to remove ARGs in animal feed. Wu et al. (2020) discovered ARGs in high-moisture corn kernels and observed a reduction in ARGs after adding *Lactobacillus brevis* [32]. In this study, the results of fluorescence quantitative PCR showed a decrease in the absolute abundance of ARGs after the addition of lactic acid bacteria, possibly due to microbial community transfer induced by lactic acid bacteria. The predominant bacteria carrying ARGs in fresh whole-crop corn include harmful bacteria or pathogens such as *Pantoea*, *Klebsiella*, *Pseudomonas*, and *Kosakonia* [33]. The natural ensiling process reduces the absolute abundance of ARGs, possibly by decreasing the levels of primary hosts *Pantoea* and *Klebsiella*. The decrease in ARGs in inoculated whole-crop corn silage might be because *Lactobacillus* dominated and suppressed pathogenic bacteria. Previous studies have shown that the application of lactic acid bacteria significantly reduces the populations of Proteobacteria and Firmicutes, known carriers of ARGs and antibiotic producers [34]. This is similar to the findings reported by Xu et al. (2021), who discovered that common pathogens *Escherichia coli* and *Staphylococcus* were significantly suppressed by *Lactobacilli* during fermentation [13].

The present study also detected whether these four lactic acid bacteria strains contain ARGs and measured the abundance of target genes within these strains [35]. The results showed that lactic acid bacteria with higher target genes increased that in whole-crop corn silage [36]. Similarly, lactic acid bacteria with lower abundance target genes reduced that in silage. It might be because different lactic acid bacteria strains contain varying numbers and types of ARGs [37]. These ARGs in silage could accumulate through gene replication or horizontal gene transfer to other bacteria. MGEs, composed of plasmids, transposons, and integrons, can promote the horizontal gene transfer of ARGs among microorganisms [38,39]. Jacobsen et al. (2007) used gnotobiotic rats as an in vivo model and found that *L. plantarum* DG 522 and *L. plantarum* DG 507 isolated from fermented dry sausages could transfer resistance genes *tet(M)* and *erm(B)* to *E. faecalis* JH2-2 through transferable plasmids [40]. Some studies also suggest that horizontal gene transfer is an important pathway for the spread of ARGs [41,42]. In this experiment, inoculating lactic acid bacteria into whole-crop corn silage reduced the abundance of transposases, with LS inoculation showing better effects, possibly because the addition of LS could reduce the abundance of ARGs located on transposases. However, while LS and E treatments decreased the abundance of integrases in whole-crop corn silage, P treatment increased its abundance, suggesting that LS and E silage inhibited horizontal gene transfer induced by integrases, whereas some ARGs in P treatment have a greater potential for horizontal transfer under integrases. Zhang et al. (2023) found that with longer ensiling times, high-abundance MGEs are associated with high potential for horizontal gene transfer [43]. Therefore, it can be inferred that *dfrK*, *acrD*, and *mexA* in whole-crop corn silage inoculated with the P treatment group may primarily undergo horizontal transfer through integrases. In conclusion, lactic acid bacteria treatment has a certain impact on HGT during the silage.

In this study, pearson correlation analysis showed that fermentation quality may be the primary driving environmental factor for changes in ARGs in whole-crop corn silage. Mantel test results indicated that the bacterial community also influenced ARGs. Previous research has found that physicochemical properties such as pH value, conductivity, moisture content, and NH_4_^+^-N in soil or compost had a positive impact on microbial communities and ARGs [44]. Similarly, the fermentation quality of silage, including pH, lactic acid, acetic acid, and propionic acid, deeply affected the abundance of ARGs in whole-crop corn silage in this study. Generally, organic acids play a crucial role as important fermentation factors during fermentation, where rapid accumulation of lactic acid can inhibit the growth of undesirable microbes and minimize nutrient loss in silage. Zhang et al. (2023) found that lactic acid content was the most significant driving factor influencing ARGs in high-moisture alfalfa silage [12]. Correspondingly, the change in ARGs in whole-crop corn silage is caused by microbial succession induced by silage quality and lactic acid bacteria, resulting in the transfer and dissemination of ARGs.

After the addition of lactic acid bacteria inoculants in whole-crop corn silage, the fermentation process was controlled by lactic acid bacteria, with Lactobacillus as the dominant genus, inhibiting pathogenic bacteria carrying ARGs. Zhang et al. (2023) found a negative correlation between the dominant genus *Lactobacillus* and ARGs belonging to lincosamide, phenicol, oxazolidinone, streptogramin, and pleuromutilin in alfalfa silage [12]. In the study by Xu et al. (2021), *Klebsiella* was identified as a potential host for oqxB and showed a positive correlation with ARGs belonging to lincosamide, phenicol, oxazolidinone, and streptogramin [13]. Zhang et al. (2023) found that Pantoea had higher abundance in fresh alfalfa, but the potential hosts in alfalfa silage (Pantoea, Pseudomonas, and Staphylococcus) decreased after inoculation with *Lactobacillus plantarum* MTD/1 or *Lactobacillus buchneri* 40788, resulting in changes in ARG abundance [43]. Notably, all MGEs were closely associated with their potential host bacteria, indicating a key role of MGEs in the horizontal transfer of ARGs. These findings above provide valuable insights into the potential hosts of the most common ARGs, yet further confirmation of hosts must be pursued through genome sequencing or the isolation of resistant gene-resistant bacterial strains.

## 5. Conclusions

In this study, a novel approach was proposed to reduce ARGs in whole-crop corn silage by inoculating lactic acid bacteria. The results indicated that ensiling could be a viable method to decrease ARGs in forages. Furthermore, the inoculation of lactic acid bacteria had a positive impact on the fermentation quality of silage, with the P treatment group demonstrating superior results. Lactic acid bacteria inoculants also decreased ARGs by inhibiting potential host bacteria. These findings suggest that adding lactic acid bacteria to whole-crop corn silage may be an effective strategy to mitigate ARG risks. To enhance feed safety in livestock farming in the future, a “perfect” lactic acid bacteria strain without ARGs should be developed to minimize ARG contamination.

## Figures and Tables

**Table 1:** Primer sequences for ARGs and MGEs in the present study.

Gene	Primer (5′→3′)	Bp	Tm
*oqxB*	TTCTCCCCCGGCGGGAAGTAC	139	60
	CTCGGCCATTTTGGCGCGTA		
*sul1*	CACCGGAAACATCGCTGCA	158	55
	AAGTTCCGCCGCAAGGCT		
*sul2*	CTCCGATGGAGGCCGGTAT	190	55
	GGGAATGCCATCTGCCTTGA		
*dfrk*	CTGCACCAGCCCCTTGAATA	211	55
	CCTGCGAGTACAAACTGGGT		
*Tn916/1545*	TCCTACAGCGACAGCCAGTGA	174	54
	TGCGTTGCTTTGGTCTGCTGGT		
*acrR*	TGGCGATCCACTCTCAGTAT	138	55
	TGTTGCACAACAGCCATTTC		
*ermB*	AAAACTTACCCGCCATACCA	139	53
	TTTGGCGTGTTTCATTGCTT		
*intl1*	CTGGATTTCGATCACGGCACG	473	60
	ACATGCGTGTAAATCATCGTCG		
*mexA*	CTCGAATTCTCCGAGGTTTCCG	143	59
	AGGATGGCCTTCTGCTTGAC		
*aac(6′)-ib-cr*	TTGCGATGCTCTATGAGTGGCTA	482	55
	CTCGAATGCCTGGCGTGTTT		
*ISCR1*	CGCCCACTCAAACAAACG	452	54
	GAGGCTTTGGTGTAACCG		

**Table 2:** Fermentation characteristics of whole-plant corn with or without LAB strains.

Item	Treatment					SEM	*p* Value
	CK	LP	P	E	LS		
Dry matter (% FW)	24.86	24.56	24.75	25.70	24.88	0.87	NS
pH	3.69 ^a^	3.63 ^b^	3.68 ^a^	3.67 ^ab^	3.66 ^ab^	0.03	<0.01
Lactic acid bacteria (lg cfu/g FW)	6.56	6.12	6.78	6.40	6.42	0.40	NS
Yeasts (lg cfu/g FW)	5.41 ^b^	5.45 ^ab^	5.38 ^b^	5.53 ^ab^	5.72 ^a^	0.21	<0.05
Mold (lg cfu/g FW)	<2.00	<2.00	<2.00	<2.00	<2.00	-	-
Coliform bacteria (lg cfu/g FW)	<2.00	<2.00	<2.00	<2.00	<2.00	-	-
Lactic acid (g/kg DM)	71.22 ^ab^	60.76 ^b^	91.35 ^a^	69.43 ^ab^	65.20 ^b^	17.79	<0.05
Acetic acid (g/kg DM)	30.90 ^ab^	24.83 ^b^	37.49 ^a^	27.74 ^ab^	26.48 ^b^	7.68	<0.05
Propionic acid (g/kg DM)	2.91 ^ab^	1.35 ^b^	7.60 ^a^	4.96 ^ab^	5.34 ^ab^	3.89	<0.05
Crude protein (g/kg DM)	83.95	85.54	85.92	85.44	85.80	3.91	NS
True protein (g/kg DM)	43.92 ^a^	40.91 ^b^	38.68 ^b^	39.84 ^b^	44.26 ^a^	2.96	<0.01
NH_3_-N (g/kg DM)	1.35 ^a^	0.89 ^b^	0.89 ^b^	1.19 ^ab^	0.89 ^b^	0.32	<0.05

DM, dry matter; NS: no significantion, FW, fresh weight; cfu, colony forming unit; SEM, standard error of means; CK, the control; LP, *Lactiplantibacillus plantarum*; P, *Pediococcus acidilactici*; E, *Enterococcus faecium*; LS, *Ligilactobacillus salivarius*; a~b, different lowercase letters in each indicator in the same row indicate significant differences at *p* < 0.05. *p* < 0.01 was a highly significant difference.

**Table 3:** Alpha diversity of bacterial communities in whole-plant corn silage with or without LAB strains.

Treatment	Sobs	Shannon	Simpson	Chao	Ace	Goods_Coverage
CK0	644.00	3.69	0.83	790.78	837.57	0.99
CK30	625.00	4.04	0.87	733.92	756.81	0.99
LP30	667.40	4.69	0.90	768.32	797.93	0.99
P30	592.40	3.99	0.84	703.86	723.74	0.99
E30	539.40	3.99	0.84	631.55	640.88	0.99
LS30	525.60	4.14	0.87	650.42	677.36	0.99

CK0, whole-crop corn before ensiling; CK30, silage without LAB strains after 30 days of fermentation; LP30, silage treated with *Lactiplantibacillus plantarum* after 30 days of fermentation; P30, silage treated with *Pediococcus acidilactici* after 30 days of fermentation; E30, silage treated with *Enterococcus faecium* after 30 days of fermentation; LS30, silage treated with *Ligilactobacillus salivarius* after 30 days of fermentation.

## Data Availability

The original contributions presented in this study are included in the article. Further inquiries can be directed to the corresponding author.
